# On the Present State of Medical Science, and the Nature and Treatment of Typhus

**Published:** 1847-12

**Authors:** Woodbride Strong


### EDITORIAL DEPARTMENT.

*On the Present State of Medical Science, and the Nature and Treatment of Typhus. A Dissertation read before the Boston Society for Medical Improvement, Sept.* 24, 1847. By WOODBRIDE STRONG, M. D.

*Communicated for the Boston Med. and Surg. Journal October* 27.

By way of introduction to the few critical remarks which will follow, we avail ourselves of this opportunity to say a word or two on the subject of criticism generally. We take it for granted that in science, as in literature, every individual has an undisputed right to the utmost latitude of opinion and taste, and should be allowed the largest liberty of expression so long as he does not interfere with the rights of others. In other words, we suppose that it will be universally conceded, that the phrase *Republic of Letters* is not merely a metaphor, but a literal fact. Criticism, like every thing else pertaining to letters, involves opinions and the expression of them, to which the utmost scope is allowable, provided the rules of propriety and courtesy are not infringed.

The last postulate is not less self evident than that preceding it, but it does not seem to be always so considered. Judging from the comments, or complaints which criticism sometimes provokes, it is a function which should be rarely exercised: should be restricted within narrow limits, or, it is a prerogative which belongs but to a few. We do not purpose to discuss this subject; we have stated our intention to offer only a word or two upon it. In venturing to assume the privileges, or the duties of criticism, we are exposed to the common reproach "who made thee a judge"? "by what right do you arrogate censorship in matters which are before the medical public? We say, in reply, that we profess to exercise no judicatory authority, nor do we presume to arrogate anything beyond the same liberty of thinking and utterance which is enjoyed by those whose writings we criticise. We claim only an equal participation in the rights and privileges belonging to every member of the Republic of Letters, when we select as themes for thinking and writing the thoughts and writings of others. Much might be said respecting the injustice and injury of curtailing the freedom of critical investigations; but we have only alluded to the subject to repudiate the idea that the self-constituted critic assumes thereby more than he has a right to assume without arrogance or egotism. He assumes the privilege of enunciating publicly his opinions, of the opinions of others, both being equally amenable to the only tribunal by which their respective claims and merits are to be adjudicated, viz., the collective opinions of the public.

With these proemial remarks, which have been suggested by the occasion, but not written with express reference to it, we proceed to the dissertation of Dr. Strong.

The Dr. speaks first of the importance of diagnosis, and is of opinion that this important branch of medical art is very much neglected. This will appear from the following quotation, in which, also, the reader will perceive that he places rather a low estimate upon medicine as exemplified in the medical literature, and medical practice of the present day:—

"Medicine has been practised with less knowledge of the subject than would suffice to do any other business, which falls to the lot of humanity. What! practise medicine, without a knowledge of the condition of the patient; guess at the disease, and guess at the remedy! This is indeed "applying remedies of which we know little, to bodies of which we know less." Who among us would commit the most trivial article worth mending, into the hands of one as ignorant of its lesions and of what is needed to repair it, as are the greater part of the profession of the diseases of their patients?

In every other occupation, the repairer of breaches first finds out the injury, the nature of the damage, and then applies himself, with a definite purpose, to repair it. The physician often, on the contrary, guesses at the injury and the remedy for symptoms without even the trouble of guessing.

D.K. 1831

This, and such as this, forms the basis of medical science; and we wonder that the world will countenance quackery, or that we in 1847, have to contend with quacks, in high places and in low, and that the long-eared public does not see the difference between us and our rivals, and respect us more. We, who have the books, the records of all the experience of the past, clothed in such panoply, have to contend with the men of yesterday; mailed knights, with naked peasantry; and the contest doubtful—victory, perching now on this, now on the other standard. We call medicine a science; and medical men, who have been systematically educated, scientific; but what claim has either to the appellation? What are the books of medicine—the best of them—we speak of therapeutics—but records of superficial views of disease, or deductions as superficial? Mere vague conjectures. What notions does every one bring to the treatment of disease, but such as these? Any one, who should pretend to certainty in this matter, would be laughed at for his presumption, or sneered at for his simplicity, as not having arrived at that esoteric state of scepticism, which marks the character of all the initiated.

The highest and the palmiest state of medicine is to be sceptical, to do really nothing, and prevent the patients doing anything; and thus to leave the case to nature. The physician, is but little better than a higher grade nurse; he has read more, and seen more, than his worthy coadjutor, who bears that name, par excellence, but he is only her senior, and has advanced one step further. She has great faith in the doctor, and his medicine; he doubts the whole matter, and considers it a humbug, a tub to the whale, which may serve to amuse him."

Such are the views of a Practitioner at Boston—the American Athens—where (as we had fondly cherished the belief) were numbered in the ranks of our honored profession as many names of those who confer honor on the profession, as in any other city of the American Union. We hope these are not the views of the *Boston Society for Medical Improvement*; and, indeed, we think we are safe in expressing doubts that a resolution to the effect that the Jacksons, Warrens, Shattucks, and Bigelows (not to mention other equal, and a host of lesser luminaries) are each "but little better than a higher grade nurse," would be adopted unanimously, by that highly respectable society. We have no right, however, to quarrel with Dr. S. for his opinion. He is at full liberty to entertain and express it, and we have no doubt there will be some to whom the statement will be cheering information—to wit—the "Quacks in high places and in low," of which he speaks.

With such views of "the present state of medical science," the inquiry naturally arises, what are the emotions of the writer? He is himself a practitioner of medicine; what are his feelings in being one of a body of men which he holds in so low estimation? It might be anticipated that so melancholy a confession would be made in a spirit of despondency, but, far from this—Dr. S. is of opinion that such an unfortunate state of things is quite unnecessary. Physicians have only to do as he does, and they will cease to be such as he represents them to be. He by no means looks upon *himself* as a "higher grade nurse." The following quotation presents the grand precept which it is his aim to enforce, illustrated by his personal example:—

I repeat, an accurate diagnosis of the intimate condition of the system is the only foundation for a scientific, a rational, or a useful treatment of disease. The treatment should be a fair deduction from such a diagnosis. Every prescription should be as legitimate an inference from a previously ascertained condition of the patient, as any conclusion in the mathematics are, from the previous steps of the process.

Such are some of my views and principles of action. In every case of disease, I have endeavored to make such a diagnosis as I have attempted, although imperfectly, to describe, and I have acted upon it boldly and decidedly, whether I had the standers-by with me or against me. Nor have I feared the unkind and censorious criticisms of my medical brethren, Not that I would purposely brave their opinions; but because I knew that what was said in the matter, was said through ignorance. I court the judgment of every fair-minded man; but must ask him, first, to understand the matter.

He, that would convince me of error in this thing, must first have gone through a course of observation such as I have been through. He must show by observation that my methods are fallacious; and not only so, but convince me that a train of proofs, as extensive as have been the observations which my eyes have seen and my other senses have recognized, have not existed—a matter of difficulty, it being no easy task to disbelieve one's own senses; when they have been most deliberately exercised."

Let the reader ponder well on the truths shadowed forth in the foregoing paragraphs! An accurate diagnosis, as a necessary preliminary for correct treatment of disease, is a maxim as novel, as it is valuable! How strange that, with all the thought bestowed upon medical subjects, this important practical injunction never before occurred to the anxious inquirer after truth!

Let every medical man, also, imitate the decision and boldness of the writer, who informs us that he endeavors, in every case, to make a diagnosis of the intimate condition of the system, and act without reference to the standers-by! We sincerly hope this idea will not be lost upon the Jacksons, the Shattucks, and other *higher grade nurses* of the city of our alma mater.

So much as preliminary to the especial theme of the dissertation, viz., Typhus or Typhoid fever. The reader will be curious to know what are the author's deductions respecting the intimate condition of the system, alias, the pathology of typhus; and what the treatment which is to be pursued "boldly and decidedly," without reference to by-standers.

The pathology is most lucidly unfolded in the following extract, which, as the reader will perceive, embraces an important pathological principle of universal application:—

"There are two elements, as has been said, which enter into every disease, unless we except those that are spontaneous—each element important to be understood, and requiring to be duly appreciated, in order successfully to combat the resulting disease, the compound of the two.

The two elements which enter into typhus, are the invading cause, and the previous condition of the system. The two combined, make up the disease which the physician is called upon to treat. There is good reason to believe that typhus of itself, appearing in a perfectly healthy body, if indeed it ever does appear in such a condition, would always be mild, and never fatal, except by accident, and that the fatal cases are owing to a complication with a previous derangement of the habit; and therefore, till this is discovered, and duly appreciated and treated, no method of practice will or can be successful,

The most constant complication is a derangement of the abdominal viscera, or costiveness. A habit of insufficient defecation is almost universal among the Yankee race ; and too prevalent among all races. In consequence there is, in some cases, an accumulation of fecal matters; and in others, an over-staying of them, and then there is a tendency to decomposition. This is kept in check by the vital energies, to a degree compatible with life, and often for a long time before it becomes predominant, when in the form of some disease it will explode. But any cause, weakening the powers of life, may disturb this nicely adjusted balance; and then the chemical affinities will become too strong for the vitality to control, and a more entire decomposition will take place. Typhus is eminently a disturbing cause; it attacks the vital principle, and prostrates all the energies of the body, and therefore tends to the result above indicated in the cases spoken of. But farther than this, it does not require any extraordinary accumulation to induce a decomposition of the contents of the bowels ; it may do it, in a condition of things compatible with high health, and in every case where there is predisposition enough to allow of the invasion of the disease."

Decomposition of the contents of the bowels is the cardinal point of the pathology and therapeutics of typhus, according to the doctrine of Dr. S. The demonstrative evidence of this is the odor emitted by the fecal evacuations. In plain anglo-saxon, the feces *stink*, ergo, they cause disease of Peyer's glands, prostration, sordes, &c. A consideration, however, here suggests itself:—Sensations are always relative. Now, as a point of departure by which to appreciate the stench of the morbid discharges of typhus, we should have been glad to have the writer's opinion as to the proper adjective to be applied to the odor of healthy feces. A medical friend of ours is wont to characterize healthy alvine evacuations as presenting a *rickness of color*, and, perhaps, by way of antithesis, Dr. S. would say, that they possess an aromatic fragrance! *Chacun a son gout!*

The practical course recommended is contained in the following:

"If the physician finds by his examinations in any kind of disease there are accumulations, over-staying of food, or a degeneration of it, the discharge of these matters is the indication; and in proportion to the length of time this has continued, and also to the degree of the degened, will be the time required to do it. Let no one expect by a single dose of medicine to repair the fault of years; many of these cases require the repetition of cathartics for weeks —some, even, for months. I have succeeded in curing cases which had resisted all previous treatment in other hands, by a daily, or almost daily, repetition of a cathartic for from six weeks to three months, and this without an accident, or weakening the patient, On the contrary, I expect, if the cathartics are well adapted, that the patient will gain strength while using them; and I have had them often gain flesh, and once at the rate of a pound a week. This happened in a child less than three years old, while he was taking a cathartic every day, which brought from three to six dejections each day. And what else should happen? Whilst you are taking away the cause that oppressed the system, the re-action must come ; as in the case of a spring, when you relieve it of a weight too heavy for its power of resistance.

How is typhus to be treated? Precisely like any other disease, on its own merits. The first busines is to examine the state of the bowels; and as the indication is found to be, so let the treatment be. If the bowels are largely, or only partially full, relieve them by the daily use of cathartics. But in typhus an obstinate state of the bowels is to be expected; and it will often happen that a week or more will pass, with the daily use of a cathartic; and with from three to six dejections each day, and that for this time the dejections will be too mucous and watery, not sufficiently fecal, after which frequent and copious fecal dejections will be obtained each day, to the speedy relief of all symptoms.

The author declares the results of this own practice as follows:

"But, aside from speculation and reasoning, the ultimate test is experience. However plausible any plan may appear, if it will not abide this test, it is valueless. In this matter I have only my own experience to appeal to, as I know of no one else, in the present or past, who has pursued the plan indicated. Since I have adopted this plan fully, which is now some eight or ten years, it has never failed me. I have not yet had a solitary case of death by typhus to report, where I have had the sole management of the case from its commencement to its termination; and what is further, I have rarely if ever had a case presenting the common phenomenon of dark sordes on the teeth, with dry and black tongue and ulcerations. Most of the cases, however threatening their aspect at first, have come, in a few days, to be so mild, and the recovery so rapid, that the parties have sometimes rather underrated their physician's attendance, looking upon his bad-tasted physic as an imposition, and the disease of too trivial a nature even to demand their thanks. And lookers-on, who have been accustomed to the disease in its ordinary form, have occasionally pronounced it impossible that a disease so mild, should be a typhus fever. I believe some parties, and those, too, where the most threatening form of the disease had been arrested, have felt that the little pittance of a bill which was presented, was made by acting on their fears, and keeping them sick, when they had some trivial disease. The plan proposed, however philosophical, however beneficial, however humane, is not one calculated to make us popular, or our patients grateful; and to those who prize popularity more than the consciousness of doing good to the unthankful, I should not recommend it."

The gist of the discourse, as respects the pathology and treatment of typhus, is, that the disease is chiefly dangerous and severe from fecal accumulations, and their decomposition; and that the aim of the practitioner should be to secure a thorough evacuation of these *materies morbi* by the persistent use of purgative remedies.

This theory and practice are far from being new. Ever since the publication of Hamilton on purgatives (a work which, with some good, we are constrained to think has done not a little harm) this class of remedies, with English and American physicians, has been more indiscriminately and extensively resorted to than any other class, and in fevers not less than in other diseases. The Abernathean doctrine, that most diseases are constitutional, and that the term constitution is synonymous with stomach and bowels, also, did much toward encouraging the use of cathartics. Indeed, it may be said that one of the characteristics of medical practice for the last half century has consisted in the excessive use of these remedies. Medicine, and, it may perhaps be added, society, are just recovering from the tendency to this therapeutical extreme. How any writer can suppose the system possesses novelty is truly surprising. That the mode "is a wide departure from that received by his medical brethren," we can well imagine to be the case, as he states it is; but that the writer should be unacquainted with the fact that it has been one of the sheet anchors of therapeutics in the estimation of not a few authors and practitioners, we could not have conceived. We can only account for it by supposing that he has himself acted upon the maxim which he inculcates, viz., that "men and not books are what need to be studied." We had supposed it to be now pretty well established, that the purgative course which he advocates is calculated to produce the prostration, dry tongue, sordes, &c.—symptoms which he declares seldom if ever occur, provided that course be resorted to. This supposition is based upon the united observations of those whose opportunities have been, individually, not less extensive than those of the author of this dissertation. Yet, the latter appeals to his experience as affording invincible evidence of the correctness of his views, for he gives us to understand that he has never lost a case of fever the treatment of which has been entirely under his supervision. Upon this evidence he rests the claims of the practice which he recommends. Now, we have no doubt that Dr. Strong is sincere in this statement, and believes it to be an honest report. We know him to be a man of high respectability, and to impeach his veracity would be the last thing to enter our mind. Nevertheless, even admitting the number of his patients to have been few, we are free to say we do not believe the statement, nor do we believe he would ever have made such a statement had he adopted the plan of *counting* propounded by Louis, of which he takes occasion to express his contempt.

But it may be asked how can a sensible, honest man deceive himself so egregiously as the skepticism you have expressed would imply? Why it is so, it may be difficult to explain, but the fact is not the less certain on that account. We were once assured by a practitioner of considerable experience that if patients with intermittent fever were bled during the hot stage, and the paroxysms arrested by quinia they would never suffer from relapses. We put the experiment into practice keeping records of the cases. It is needless to say that it was speedily falsified. This instance occurs to us. Cannot our readers recall similar examples of self-deception? Nothing is more common in intercourse with practitioners than to hear the most positive statements enunciated with respect to the invariable efficacy of certain modes of practice in certain diseases, but the same results seldom follow similar practice in the hands of those thereby induced to adopt it. Hence, the immense advantage of the numerical system in enabling us to avoid errors, and in giving precision and certainty to medical experience.

There never was a truer remark than that "medicine has suffered more from false facts than from false theories." Theories may often be safely adopted or rejected on their abstract merits, but false facts, supported by positive testimony, appeal not so much to reason, as to faith. The liabilities to self-delusion, and the difficulties in the way of determining facts, owing to the number and complexity of the elements which they involve, are not enough considered. We are too apt to receive them without hesitation or allowance, and such is the tenacity with which the mind adheres to what have been acknowledged to be facts, that their correction requires much time and effort when they have once obtained currency. The history of medicine furnishes an abundance of striking illustrations of the flexibility of medical experience. What doctrine, however absurd, but was professedly sustained by the results of experience! Brunonism, on the one hand, and Broussaism, on the other, antipodes of practice, each boasted that it was only necessary to witness its influences in relieving disease, and diminishing mortality, to be convinced of its verity. Homœopathy, with its amazing absurdities, appeals most confidently to facts, and there are some honest persons who imagine that they have seen with their own eyes triumphant exhibitions of its power over disease! Returning to the dissertation, and waiving discussion of the pathological and therapeutical merits of the views which it sets forth, we conclude by expressing the hope that those readers who deem them deserving of attention, will be content to try them by the experience of the past, by conflicting facts furnished by the testimony of those whose observations lead to opposite conclusions, before resorting to the test of experimental inquiry. We must confess we have some bowels of compassion for the bowels of the present and rising generation, and we would save them the painful duty of demonstrating anew, in this instance, the truth of the maxim *experientia fallax*.

